# Transcriptional regulator Taf14 binds DNA and is required for the function of transcription factor TFIID in the absence of histone H2A.Z

**DOI:** 10.1016/j.jbc.2022.102369

**Published:** 2022-08-13

**Authors:** Kadri Peil, Signe Värv, Ivar Ilves, Kersti Kristjuhan, Henel Jürgens, Arnold Kristjuhan

**Affiliations:** 1 Institute of Molecular and Cell Biology, University of Tartu, Tartu, Estonia; 2 Institute of Technology, University of Tartu, Tartu, Estonia

**Keywords:** transcription, Taf14, Taf2, H2A.Z, TFIID, preinitiation complex (PIC), chromatin, NDR, nucleosome-depleted region, PIC, preinitiation complex, TFIID, transcription factor IID, TFIIF, transcription factor IIF

## Abstract

The transcriptional regulator Taf14 is a component of multiple protein complexes involved in transcription initiation and chromatin remodeling in yeast cells. Although Taf14 is not required for cell viability, it becomes essential in conditions where the formation of the transcription preinitiation complex is hampered. The specific role of Taf14 in mediating transcription initiation and preinitiation complex formation is unclear. Here, we explored its role in the general transcription factor IID by mapping Taf14 genetic and proteomic interactions and found that it was needed for the function of the complex if Htz1, the yeast homolog of histone H2A.Z, was absent from chromatin. Dissecting the functional domains of Taf14 revealed that the linker region between the YEATS and ET domains was required for cell viability in the absence of Htz1 protein. We further show that the linker region of Taf14 interacts with DNA. We propose that providing additional DNA binding capacity might be a general role of Taf14 in the recruitment of protein complexes to DNA and chromatin.

Initiation of transcription is a complex process requiring coordinated assembly of the preinitiation complex (PIC) to gene promoters. Once fully formed, PIC covers about 80 to 200 base pairs of DNA (1) and it is evident that the accessibility of promoter DNA is crucial in this process. In eukaryotic cells, access to DNA is hindered by chromatin structure and multiple mechanisms have evolved to open promoter chromatin for transcription initiation. One of these mechanisms is the maintenance of nucleosome-depleted regions (NDRs) in promoter DNA (2, 3). In yeast, the NDRs are flanked with nucleosomes where the canonical histone H2A is substituted with Htz1, a yeast homolog of metazoan histone H2A.Z (4, 5, 6, 7). In general, the Htz1 nucleosomes cover the transcription start sites of genes and are displaced from the promoters during the initiation of transcription (8, 9). It has been shown that the promoter nucleosomes tend to be “fragile”, *i.e*., they disassemble more easily than other nucleosomes (10), supporting the observation that H2A.Z can modulate the stability of the nucleosome (11). Despite its apparent role in promoter nucleosomes, Htz1 is nonessential in yeast, and the growth rate of *htz1Δ* strains is very similar to that of wildtype cells. However, activation of inducible genes is delayed in *htz1Δ* cells, emphasizing the role of Htz1 in the regulation of promoter accessibility and transcription initiation (5, 12).

The formation of PIC is initiated by a general transcription factor IID (TFIID), which binds the promoter first and forms the core of PIC (13, 14). TFIID consists of the TATA-binding protein and its associated factors (TAF proteins). The TFIID complex is found in all eukaryotes, although some variations in the number and size of TAF proteins exist in different species (15). In *Saccharomyces cerevisiae,* there are 14 TAF proteins, and compared to other eukaryotes, yeast has an extra constituent—Taf14. It is the only nonessential component of TFIID and is also a subunit of several other protein complexes involved in chromatin remodeling (INO80, SWI/SNF, RSC), histone acetylation (NuA3), or transcription initiation (transcription factor IIF [TFIIF]). Although deletion of *TAF14* is tolerated in yeast cells, it leads to several phenotypes including slow growth on rich media, sensitivity to heat, and various genotoxic agents (16, 17, 18, 19, 20). Taf14 interacts with TFIID *via* its largest subunit, Taf2. The C terminus of Taf2 contains two separate regions that can recruit Taf14 (Fig. 1*A*). However, the presence of Taf14 is not critical for TFIID as the strains expressing C-terminally truncated Taf2 grow nearly as well as wildtype cells (21).

**Figure 1:**
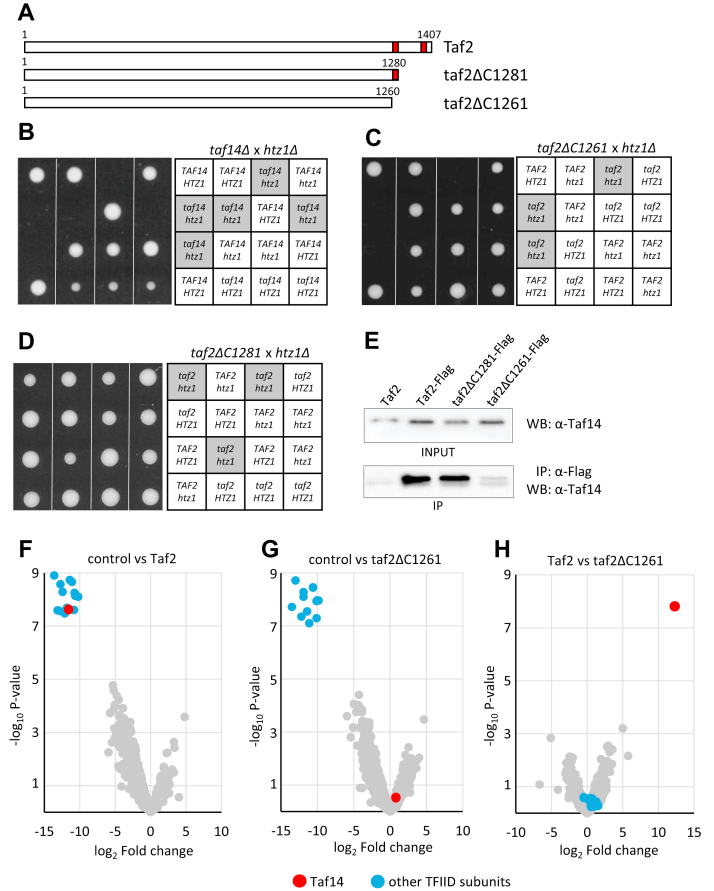
**Deletion of HTZ1 is lethal in *taf14Δ and taf2ΔC1261* cells.***A,* scheme of Taf2 proteins with corresponding amino acid residue numbers. Red boxes indicate the locations of Taf14 binding sites on Taf2 protein. *B-D,* tetrad analysis of the progeny from the crosses between *htz1Δ* and *taf14Δ* strains (*B*), *htz1Δ* and *taf2ΔC1261* strains (*C*), and *htz1Δ* and *taf2ΔC1281* strains (*D*). The tetrads were dissected on YPD plates and photographed after 3 days of growth at 30 °C. Four tetrads from each dissection are shown. Legends of genotypes are shown on the right panels. *E,* Flag-tagged full-length Taf2 and its C-terminally truncated proteins were immunoprecipitated, and the presence of coprecipitated Taf14 protein was analyzed with an anti-Taf14 polyclonal antibody. Representative immunoblot from three biological replicates is shown. *F-H,* Mass spectrometry analysis of proteins coprecipitated with full-length Taf2 and Taf2ΔC1261. The volcano plots represent a pairwise comparison of nontagged strain (control) *versus* Taf2-Flag (*F*); nontagged strain *versus* Taf1ΔC1261-Flag (*G*); and Taf2-Flag *versus* Taf1ΔC1261-Flag (*H*). Taf14 and other TFIID subunits are color-marked. All samples were analyzed in triplicates. TFIID, transcription factor IID.

Two structural domains have been defined in Taf14 protein. The YEATS domain (**Y**af9, **E**NL, **A**F9, **T**af14, and **S**as5) is located in the N-terminal part of the protein and the ET (extra-terminal) domain is located in the C terminus (22, 23). These domains are connected by an unstructured linker region (Fig. 2*A*). The YEATS domain is an evolutionarily conserved protein structure that mediates Taf14 interaction with acyl-modified histone N-terminal tails (24, 25), while the ET domain is required for the incorporation of Taf14 into protein complexes (17, 21, 23, 26, 27). The functions of Taf14 are not entirely clear. It has been proposed that the YEATS domain supplements Taf14-containing complexes with an additional acyl-lysine binding activity, helping their localization to the specific chromatin regions (24, 25, 28, 29). However, expression of YEATS-less Taf14 protein in *taf14Δ* cells rescues the majority of *taf14Δ* phenotypes, indicating that the YEATS-independent roles of Taf14 might be even more critical for its activity (17, 23, 30).

**Figure 2:**
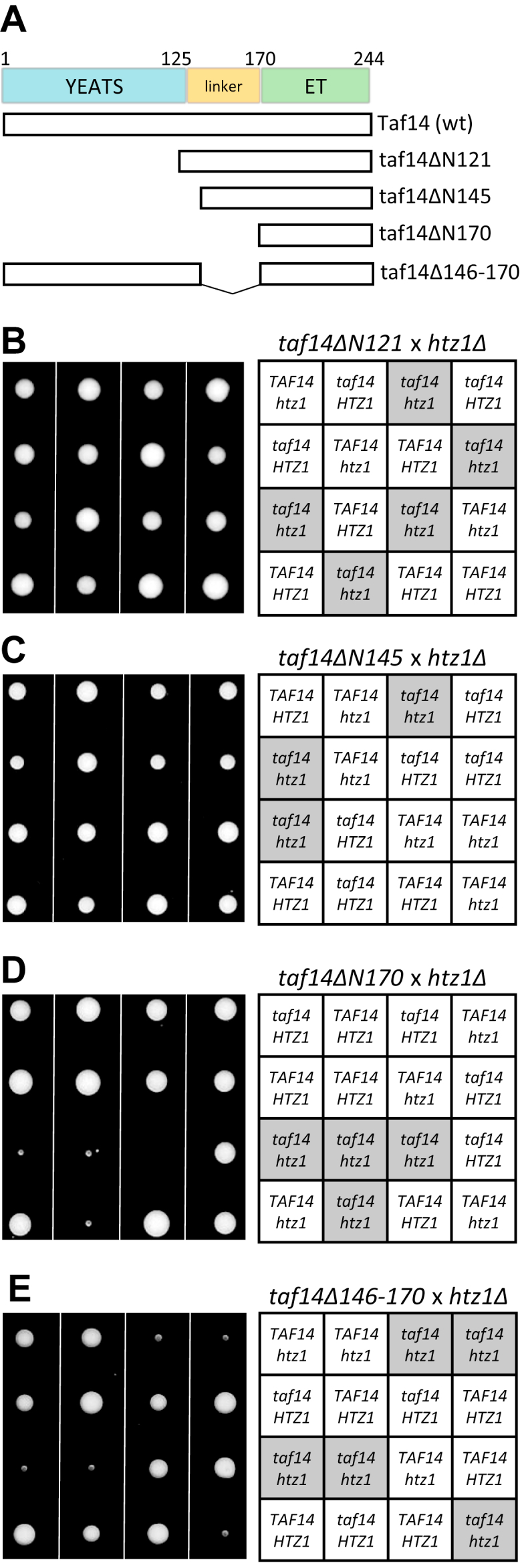
**Genetic interactions of Taf14 deletion mutants with *htz1Δ.****A,* scheme of Taf14 proteins. The locations of YEATS, linker, and ET domains with corresponding amino acid residue numbers are indicated. *B-E,* tetrad analysis of the progeny from the crosses between *htz1Δ* and *taf14ΔN121* strains (*B*), *htz1Δ* and *taf14ΔN145* strains (*C*), *htz1Δ* and *taf14ΔN170* strains (*D*), *htz1Δ* and *taf14Δ146-170* strains (*E*). The tetrads were dissected on a YPD medium, and plates were photographed after 3 days of growth at 30 °C. Four tetrads from each dissection are shown. Legends of genotypes are shown on the right panels.

We explored the functions of Taf14 by studying its role in the TFIID complex. We found that Taf14 becomes essential for TFIID in strains, where the histone variant Htz1 is missing. Mapping the potential functional domains and activities of Taf14 revealed that it also contains a DNA-binding region, which turned out to be essential for the TFIID complex in the *htz1Δ* strain background.

## Results

### Taf2–Taf14 interaction is essential for the viability of htz1Δ cells

While Taf14 is a subunit of several protein complexes, it can be selectively excluded from TFIID by deletion of the Taf2 C-terminal domain—the binding site of Taf14 in the TFIID complex. To explore whether Taf14 might have a more pronounced effect in conditions where the normal nucleosomal structure in promoter regions is distorted, we tested *TAF14* genetic interaction with *HTZ1*. We found that simultaneous deletion of *TAF14* and *HTZ1* led to synthetic lethality, suggesting that Taf14 becomes essential in the absence of Htz1 (Fig. 1*B*). To explore whether TFIID can function without the Taf14 subunit in the *htz1Δ* background, we crossed *htz1Δ* cells with the strain expressing C-terminally truncated Taf2 (*taf2ΔC1261;* Fig. 1*A*) and found that also *taf2ΔC1261 htz1Δ* cells were inviable (Fig. 1*C*). The latter result was surprising, as the growth rates of strains carrying either *taf2ΔC1261* or *htz1Δ* mutations alone were similar to the wildtype strain. Htz1 protein is incorporated into chromatin by the SWR1 chromatin remodeling complex, and deletion of its subunits Swr1 or Swc5 results in complete loss of Htz1 from nucleosomes (8, 31, 32, 33). We combined deletions of *SWR1* or *SWC5* genes with *taf2ΔC1261* truncation and found that these combinations were also lethal, confirming that the presence of Htz1 in chromatin was required for the survival of *taf2ΔC1261* cells (Fig. S1). Together, these results indicate that the Taf2 C-terminal domain and Htz1 may play essential and overlapping roles in cells, presumably during the early steps of PIC formation.

It has been shown that Taf2 C terminus contains two separate domains that bind Taf14, and the deletion of these disrupts Taf14 interaction with Taf2 (21). To study whether the Taf2–Taf14 interaction is essential to rescue synthetic lethality with *HTZ1* deletion, we made a yeast strain where the proximal Taf14 binding site on Taf2 remained intact, while the rest of the Taf2 C-terminal domain was removed (*taf2ΔC1281;* Fig. 1*A*). We confirmed that the removal of both Taf14 binding sites from Taf2 protein (Taf2ΔC1261) efficiently disrupts its interaction with Taf14 *in vivo*, while the Taf2 mutant retaining a single Taf14 binding site (Taf2ΔC1281) is fully competent for Taf14 binding (Fig. 1*E*). Tetrad analysis of the progeny of *HTZ1/htz1Δ TAF2/taf2ΔC1281* strain revealed that *taf2ΔC1281* was tolerated in the *htz1Δ* background (Fig. 1*D*). This result shows that Taf14 recruitment to the TFIID complex becomes essential for viability when Htz1 is absent.

To test whether Taf2 C terminus is interacting also with any other proteins apart from Taf14, we affinity-purified wildtype and C-terminally truncated Taf2 from yeast strains and identified the copurified proteins by mass spectrometry analysis. The identical set of proteins was copurified with both proteins, and all the significant interactions were detected with other TFIID subunits. The only exception was Taf14, which was recovered in the complex with Taf2, but not with the Taf2ΔC1261 protein (Fig. 1, *F*–*H*). This confirms that C-terminal truncation of Taf2 disrupts Taf14 binding to the TFIID complex, while no other protein–protein interactions are affected.

### The linker region of Taf14 is required for the rescue of synthetic lethality with htz1Δ

It has been shown that the Taf14 YEATS domain interacts with acetylated and crotonylated histone H3 N-terminal tails *in vitro*, suggesting its role in targeting Taf14-containing protein complexes to modified nucleosomes (24, 25, 28, 29). On the other hand, the slow-growth phenotype of *taf14Δ* cells can be rescued by the expression of truncated Taf14 protein that lacks the entire YEATS domain, indicating that YEATS-independent functions of Taf14 play a major role in the suppression of *taf14Δ* phenotype (17, 23, 30). To test which domains of Taf14 are required for the suppression of *htz1Δ taf14Δ* lethality, we examined the survival of Taf14 deletion mutants in the *htz1Δ* background (Fig. 2*A*). We found that both *taf14ΔN121 htz1Δ* and *taf14ΔN145 htz1Δ* cells were viable, indicating that the YEATS domain and the N-terminal part of the linker region of Taf14 were not required to suppress the synthetic lethality with *HTZ1* deletion (Fig. 2, *B* and *C*). However, when both the YEATS and the entire linker domain were deleted, the growth of cells was severely affected as only microcolonies of *taf14ΔN170 htz1Δ* mutants were observed (Fig. 2*D*). Finally, to confirm that the linker region of Taf14 was essential for the survival of *htz1Δ* cells, we made an internal deletion of Taf14 protein removing its amino acids 146-170. As expected, the growth of *taf14Δ146-170 htz1Δ* was also severely affected, underlining the importance of the Taf14 linker region in the proteins’ function (Fig. 2*E*). Notably, none of the Taf14 deletion mutants showed growth defects in *HTZ1* cells, indicating that no other genetic interactions were affected by the Taf14 mutants. We concluded that both linker and ET domains of Taf14 were required for cell survival in the *htz1Δ* strain background, while the Taf14 protein expressing only the ET domain was unable to suppress synthetic lethality with *htz1Δ*.

### Protein interactions of taf14ΔN170 and wildtype Taf14 are identical

Taf14 interacts with multiple protein complexes *via* its ET domain (17, 21, 23, 26, 27), and it has been suggested that Taf14 might form homodimers (34). Both functions may be important for Taf14 to rescue cell lethality in *htz1Δ* strain background. For instance, the Taf14ΔN170 protein might be unable to interact with some specific protein complex that normally contains Taf14 as its subunit. Also, possible dimerization of Taf14 could mediate interactions between different protein complexes, which may become critical in the absence of Htz1.

**Figure 3:**
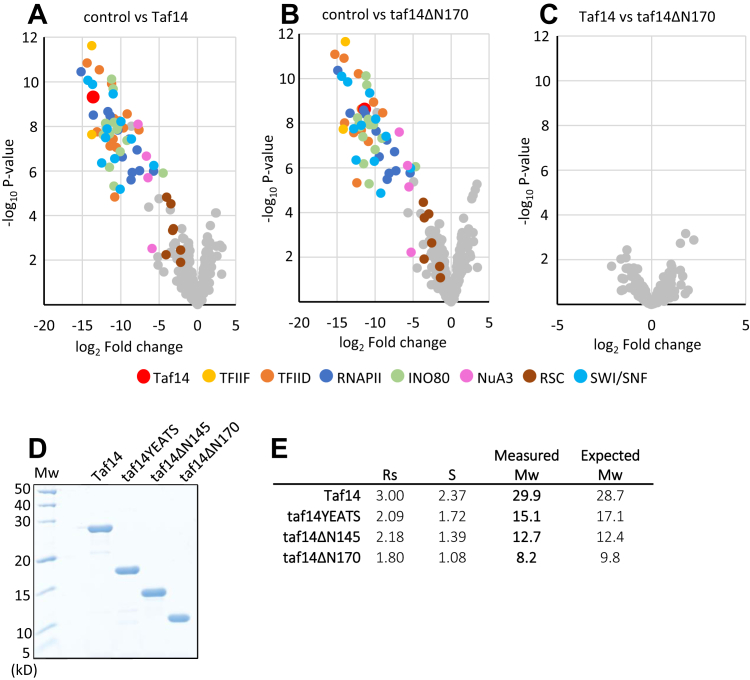
**Protein interactions of Taf14.***A-C,* mass spectrometry analysis of proteins coprecipitated with full-length Taf14 and Taf14ΔN170 proteins. The volcano plots represent a pairwise comparison of nontagged strain (control) *versus* Flag-Taf14 (*A*); nontagged strain *versus* Flag-Taf14ΔN170 (*B*); and Flag-Taf14 *versus* Flag-Taf14ΔN170 (*C*). Taf14 and the subunits of Taf14-containing protein complexes are color-coded in panels A and B. All samples were analyzed in triplicates. *D*, Coomassie Brilliant Blue stained Tris-tricine SDS-polyacrylamide gel (16%) showing the purified Taf14 proteins. *E*, determined Stokes radiuses (Rs) and Svedberg sedimentation coefficients (S) of the Taf14 proteins together with the measured apparent molecular weights (Mw) of the respective proteins. The expected Mw of monomeric proteins is shown next to each measured Mw value. The results of size exclusion chromatography and glycerol gradient sedimentation analyses are shown in Fig. S2. RNAPII, RNA polymerase II; TFIID: transcription factor IID; TFIIF: Transcription factor IIF.

We used affinity purification coupled with mass spectrometry analysis to determine the proteins that coprecipitated with wildtype Taf14 and Taf14ΔN170. Both proteins efficiently coprecipitated all subunits of known Taf14-containing complexes TFIID, TFIIF, INO80, SWI/SNF, and NuA3. Also, some subunits of the RSC complex were coprecipitated, although in lesser quantities than the subunits of other Taf14-interacting complexes (Fig. 3, *A* and *B*). In concordance with previous studies, we also detected all subunits of the RNA polymerase II in Taf14 precipitations, likely indicating tight interaction of TFIIF and RNA polymerase II (35, 36). However, we did not detect any significant difference in the set of proteins interacting with full-length Taf14 or Taf14ΔN170, confirming that no other regions than the ET domain of Taf14 are required to mediate its interaction with protein complexes (Fig. 3*C*).

To test the possible dimerization of Taf14, we purified the full-length and truncated versions of Taf14 proteins in *E. coli* (Fig. 3*D*) and determined their Stokes radiuses (R_s_) and Svedberg sedimentation coefficients (S) by size exclusion chromatography and glycerol gradient sedimentation analysis, respectively (Fig. S2). We used these data to calculate the apparent molecular weight of the proteins (37) and found that all Taf14 proteins were purified as monomers (Fig. 3*E*). This confirms that the oligomerization status of full-length Taf14 was not different from the ΔN170 protein and suggests that Taf14 is unlikely a direct mediator of interactions between protein complexes. Together, these results show that Taf14ΔN170 protein retains all appropriate protein–protein interactions of full-length Taf14, but this is insufficient to suppress its synthetic phenotype with *htz1Δ*.

### Taf14 provides an additional DNA binding domain to the TFIID complex

**Figure 4:**
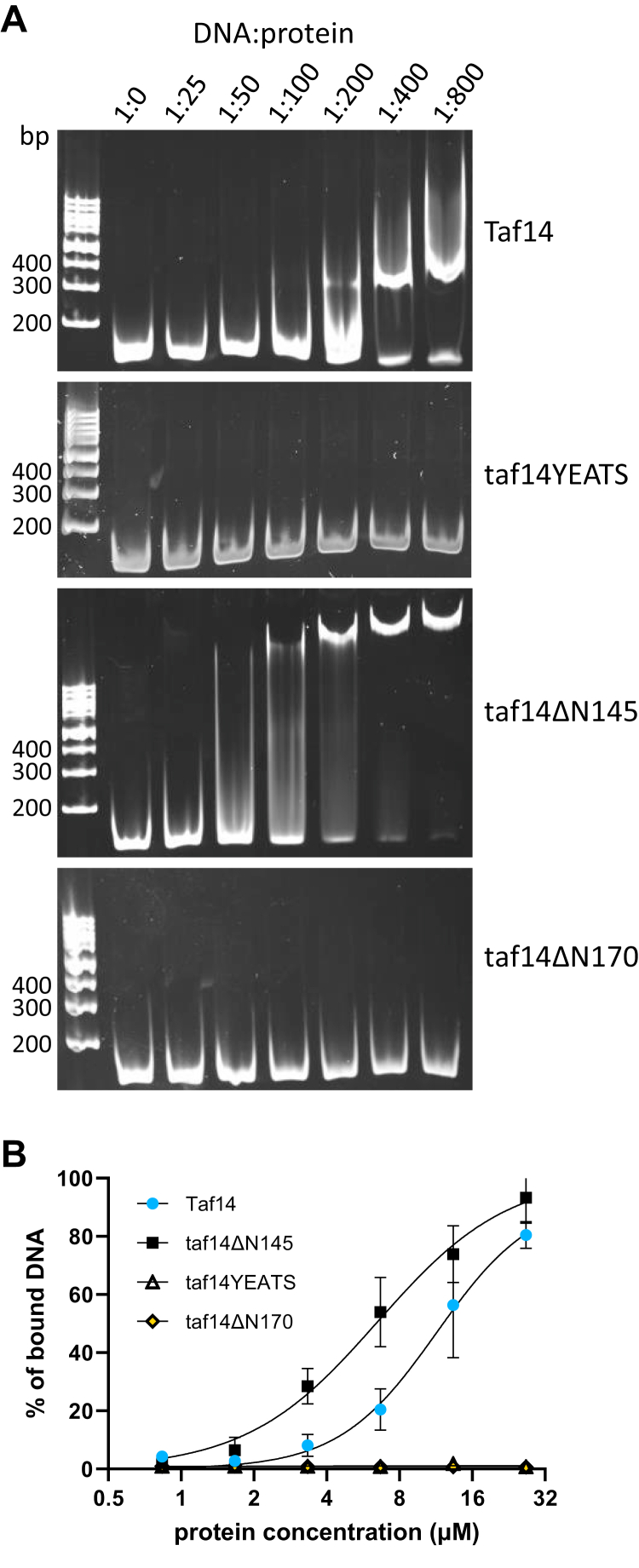
**Taf14 linker region is required for DNA binding.***A*, DNA electrophoretic mobility shift assay with purified Taf14 proteins. Each reaction contained 0.5 pmol dsDNA together with increasing amounts of purified Taf14 proteins (DNA to protein molar ratios are shown on top). A representative image of three replicates is shown. *B,* average binding curves of wildtype and mutant Taf14 proteins to dsDNA ligand. The fraction of shifted DNA was quantified for each lane and plotted against protein concentration. Error bars show the standard deviations (n = 3).

As the recruitment of Taf14 to the TFIID complex is vital in *htz1Δ* cells, we hypothesized that Taf14 may be particularly required when the accessibility of promoters is decreased. For example, TFIID may need additional assistance to compete with nucleosomes and establish contacts with promoter DNA. To test whether Taf14 might possess a DNA-interaction activity, we used the purified Taf14 proteins in DNA binding assay. Both full-length Taf14 and Taf14ΔN145 proteins bound DNA efficiently, while the mutants lacking the linker region (Taf14YEATS and Taf14ΔN170) did not (Fig. 4*A*). This shows that the linker region between amino acid residues 146 and 170 is required for DNA binding of Taf14 protein, while the YEATS domain is dispensable for this activity. The apparent dissociation constant values for DNA binding by Taf14 and Taf14ΔN145 were in the low micromolar range, suggesting nonspecific DNA binding by Taf14 (Fig. 4*B*). Taken together, our results revealed that from all the tested possible contributions of Taf14 to the functions of TFIID, only the DNA binding activity was missing from the Taf14ΔN170 mutant compared to the wildtype protein. We thus propose that the main role of Taf14 in the TFIID is to provide an additional DNA binding domain to the complex, which becomes essential in conditions where the NDR maintenance is not supported by Htz1.

## Discussion

Initiation of transcription in higher eukaryotes is a complex process that begins with the formation of PIC and requires the binding of TFIID to gene promoters. In yeast, TFIID consists of TATA-binding protein and 14 TAF proteins of which Taf14 is the only nonessential one. To understand the role of Taf14 in the TFIID complex, we used yeast strains expressing C-terminally truncated Taf2 protein. Deletion of the Taf2 C terminus disrupts Taf2–Taf14 interaction and therefore abolishes Taf14 incorporation into TFIID (21). Although the deletion of the Taf2 C terminus did not cause growth defects in yeast cells, it became essential for viability in the *htz1Δ* background (Fig. 1*C*). We confirmed that upon the deletion of the Taf2 C terminus, Taf14 was the only protein missing from the TFIID complex, suggesting that Taf14 became essential for TFIID in the *htz1Δ* background (Fig. 1, *F*–*H*). Indeed, when we reconstructed the Taf2–Taf14 interaction, the synthetic lethality of truncated Taf2 in *htz1Δ* cells was rescued (Fig. 1*D*). Next, we dissected the regions of Taf14 essential for cell survival in the *htz1Δ* background and found that the YEATS domain was dispensable, while the linker region together with the ET domain was required for viability (Fig. 2). We found that the ET domain alone was sufficient to establish all Taf14-specific protein–protein interactions (Fig. 3), but it was unable to rescue the *htz1Δ taf14Δ* phenotype. This suggested that the linker region provided Taf14 an additional function that became essential in the *htz1Δ* background. The Taf14 linker region is rich in positively charged amino acid residues, suggesting that it could make electrostatic interactions with the negatively charged phosphodiester backbone of DNA. Therefore, we hypothesized that the Taf14 linker domain might interact with DNA and provide an additional DNA binding module to the TFIID complex. We tested this hypothesis *in vitro* with purified proteins and found that the linker region was indeed required for a nonspecific DNA binding of Taf14 (Fig. 4). Together, these results show that in addition to the YEATS and ET domains, Taf14 has an additional functional region, which mediates its interaction with DNA. In line with our results, the DNA-binding activity of the Taf14 linker region was also identified in a recent study proposing that Taf2–Taf14 interaction induces a conformational shift in Taf14 and enhances its DNA binding capacity (38). Interestingly, also mammalian YEATS-domain containing protein AF9 binds DNA (28), suggesting that dual binding to histones and DNA might be a common feature of the YEATS proteins. While in the case of AF9, the YEATS domain binds both the histone tails and DNA, these activities are separated in Taf14. A comparison of the surface electrostatic potentials of AF9 and Taf14 YEATS domains revealed that one side of the AF9 YEATS domain contains a patch of positively charged amino acid residues, similar to what is present in the Taf14 linker region, but absent in the Taf14 YEATS domain (28, 38). This suggests that both proteins might bind DNA by the same mechanism, which is further supported by the similarity of their DNA binding affinities (Kd-s in the low micromolar range).

Considering that Taf14 contains both acyl-lysine binding and DNA binding activities and is a common subunit of multiple protein complexes, we propose that it might be a universal auxiliary module, assisting protein complexes in binding to chromatin and DNA. In TFIID, Taf14 might participate in the initial binding of the complex to promoter DNA. In normal conditions, where NDRs are efficiently maintained, the effect of Taf14-mediated DNA interaction might be negligible, but it becomes crucial in *htz1Δ* cells, where TFIID is forced to contend with more condensed nucleosomal structures. Notably, as Taf14 is also a subunit of TFIIF, it is not completely absent from the fully formed PIC, even if it is expelled from TFIID. This suggests that in *htz1Δ* cells, Taf14 becomes essential during the early steps of PIC formation when TFIID binds promoters and initiates the recruitment of other general transcription factors. One intriguing possibility is that instead of enhancing the DNA binding capacity to TFIID, the primary function of Taf14–DNA interaction might be stabilizing nucleosome-free DNA in promoter regions and serving it as an assembly site for other PIC components. This would also explain the apparent redundancy of Taf2–Taf14 interaction with the presence of Htz1 in chromatin—in both ways the promoter DNA is made more accessible for PIC formation, either due to Taf14-mediated presentation of free DNA or due to relatively easy disassembly of the Htz1-containing nucleosomes. At least one of these mechanisms must be functional for cell survival, while the wildtype cells can utilize both pathways simultaneously.

## Experimental procedures

### Construction of yeast strains and tetrad analysis

All yeast strains were in the W303 background and are listed in Supplementary Table 1. In the strains expressing deletion mutants of Taf2 or Taf14 proteins, the corresponding endogenous genes were replaced with genes encoding either N-terminally 3xFlag-tagged Taf14 or C-terminally 3xFlag-tagged Taf2 proteins. The *TAF14* mutant strains express the following parts of the protein: ΔN121—amino acids 122-244; ΔN145—amino acids 146-244; ΔN170—amino acids 171-244; and Taf14YEATS—amino acids 1-138. The *TAF2* mutant strains express the following parts of the protein: ΔC1261—amino acids 1-1260; ΔC1281—amino acids 1-1280. Tetrad dissection was performed with the Singer MSM System 300 dissection microscope on YPD medium plates, photographed after 3 days of growth at 30 °C, and replica-plated to selective media for confirmation of 2:2 segregation of the marker genes and identification of the genotypes of colonies. At least 40 tetrads were dissected from each crossing.

### Co-immunoprecipitation

Taf2 co-immunoprecipitations were performed using strains AKY1900, AKY2062, and AKY2500 expressing the C-terminally 3xFlag-tagged Taf2 wildtype or mutant proteins. Cell pellets from 50 ml exponentially grown cells were resuspended in 1 ml of lysis buffer (50 mM Hepes pH7.5, 150 mM NaCl, 1 mM EDTA, 10% glycerol, 0.5% Triton X-100, 0.5 mM DTT and 1× Roche protease inhibitor cocktail) and mechanically sheared in the presence of glass beads. After 5 min centrifugation at 16,000 g, 1 ml of supernatant (whole cell lysate) was incubated with 20 μl anti-FLAG M2 agarose beads (Sigma-Aldrich; A2220) at 4 °C for 2 h. Beads were washed three times with WB1 (50 mM Hepes pH 7.5, 150 mM NaCl, 1 mM EDTA, 5% glycerol, 0.5% Triton X-100, 0.5 mM DTT) and one time with WB2 (50 mM Hepes pH7.5, 150 mM NaCl, 1 mM EDTA, 5% glycerol). Beads were resuspended in 30 μl 2XSDS sample buffer (125 mM Tris-HCl ph 6.8, 4% SDS, 10% 2-mercaptoethanol, 20% glycerol, 0,004% bromophenol blue), incubated for 5 min at 95 °C, and vortexed briefly. Immune complexes were separated on 15% SDS-polyacrylamide gel, and proteins were detected by immunoblotting using rabbit polyclonal anti-Taf14 (A278, antibodies.com) or mouse monoclonal ANTI-FLAGM2 (Sigma-Aldrich; F3165) primary antibodies. Anti-mouse or anti-rabbit HRP-conjugated secondary antibody and ImmobilonWestern Chemiluminescent HRP substrate (Millipore; WBKLS0500) were used for signal visualization on ChemiDocXRS^+^/ImageLab Software (Bio-Rad). A sample from the AKY152 strain expressing untagged proteins was used as a negative control. All co-immunoprecipitation experiments were repeated in three biological replicates.

### Mass spectrometry analysis

Frozen yeast cell pellets harvested from a 50 ml exponentially growing shaker culture were thawed in 1 ml of lysis buffer (25 mM Hepes-KOH pH 7.6, 50 mM KCl; 0.02% Tween-20, 10% glycerol, 1 mM EDTA, 0.4 mM PMSF, 2 mM β-mercaptoethanol, 0.1% NP-40, 2 mM MgCl_2_, 2 mM β-glycerophosphate, 2 mM NaF). The cells were homogenized with glass beads in a Precellys 24 tissue homogenizer (Bertin Technologies), the benzonase nuclease (Millipore) was added (50 U/ml for Taf2, 250 U/ml for Taf14 IP-MS), and the extract was incubated for 40 to 60 min at 4 °C with end-over-end mixing to completely digest the nucleic acids. KCl concentration was adjusted to 250 mM, and the extract was incubated for an additional 20 min with end-over-end mixing at 4 °C before clearing by centrifugation. The cleared extracts were incubated with 15 μl of packed anti-FLAG M2 agarose beads (Sigma-Aldrich; A2220) for 4 h at 4 °C with end-over-end mixing. The beads were washed six times with 1 ml of 250C buffer (25 mM Hepes-KOH pH 7.6, 250 mM KCl, 0.02% Tween-20, 10% glycerol, 1 mM EDTA, 0.4 mM PMSF, 2 mM β-mercaptoethanol, 2 mM β-glycerophosphate, 2 mM NaF), collecting the beads by centrifugation after each wash step. The beads were transferred to Bio-Rad mini spin columns, and the bound protein complexes were eluted with 3 x 40 μl pH 2.5 glycine buffer (50 mM glycine-HCl, 150 mM NaCl), adding 4 μl of 1M Tris-HCl pH 8.0 to each eluted fraction. The fractions were pooled at the end, and proteins precipitated overnight with TCA at 20% final concentration at 4 °C. This was followed by centrifugation, washing with -20 °C acetone, and air drying of the protein pellets. Trypsin digestion of the precipitated proteins and their mass spectrometric analysis was carried out by the proteomics core facility of the Institute of Technology, University of Tartu, following the protocol described previously (39). The digested peptides were separated on 3 μm ReproSil-Pur C18AQ 15 cm × 75 μm ID emitter-column (New Objective) attached to an Agilent 1200 series nano-LC and detected with an LTQ Orbitrap XL (Thermo Fisher Scientific) mass spectrometer. The proteomics datasets preprocessed with MaxQuant were further analyzed with the LFQ-Analyst software (Monash University) (40).

### Protein purification and size exclusion chromatography

Genes encoding full-length, Taf14YEATS, Taf14ΔN145, and Taf14ΔN170 proteins with N-terminal strep affinity tag were inserted into the pET28 expression plasmid between the NcoI and SacI cloning sites. The plasmids were transformed into *E. coli* BL21 (DE3) cells, which were induced to express the respective proteins with 0.3 to 0.5 mM IPTG in a 1L shaker culture volume at the cell density (*A*_600_) of approximately 0.8 to 1.0. Cells were collected after shaking for 18 h at 18°C, washed once with PBS, and resuspended in 25 ml 500N buffer (25 mM Hepes-NaOH pH 7.6, 500 mM NaCl, 10% glycerol) supplemented with 1 mM DTT and 1× Roche protease inhibitors mix without EDTA. The cell suspension was frozen in liquid nitrogen and stored at -80 °C. For purifying the expressed proteins, the cells were thawed, treated with 1 mg/ml lysozyme for 30 min at 4 °C, and sonicated with 3 x 40 s bursts of the Bandelin SONOPULS ultrasonic homogenizer. The resulting extract was cleared by centrifugation and passed through the Strep-Tactin XT four flow (IBA Lifesciences GmbH) column. The column was washed four times with N500 buffer supplemented with 0.2 mM PMSF and twice with N100 buffer (25 mM Hepes-NaOH pH 7.6, 100 mM NaCl, 10% glycerol). Bound protein was eluted with N100 supplemented with 50 mM biotin. The eluate was concentrated in Amicon Ultra-4 centrifugal filter units and injected into the Superdex 75 10/300 GL SEC column attached to the GE Healthcare ÄKTAmicro chromatography system. The column was developed with N100 buffer, and 0.5 ml fractions of the purified protein peak were collected. To determine the Stokes radius of the purified Taf14 proteins, the Superdex 75 10/300 GL SEC column was calibrated with the following protein standards: bovine serum albumin (Rs = 3,5), chicken ovalbumin (Rs = 3,05), bovine carbonic anhydrase (Rs = 2,1), and bovine ribonuclease A (Rs = 1,64).

### Sedimentation analysis of the Taf14 proteins

Purified Taf14 proteins were mixed with the following protein standards with known Svedberg coefficients: chicken conalbumin (S20,w =5,05), chicken ovalbumin (S20,w =3,66), bovine carbonic anhydrase (S20,w =2,8), and bovine ribonuclease A (S20,w =2,0) (41). Four milliliter 5 to 10% linear glycerol gradients in a 25 mM Hepes-NaOH pH 7.6, 100 mM NaCl buffer were prepared using a GE Healthcare ÄKTAmicro chromatography system, and 100 μl of the Taf14 + standard protein mix was loaded on top of each gradient. Centrifugation was carried out in a Beckman Optima L90-K ultracentrifuge in an SW55 swinging-bucket rotor at 180,000 g for 24 h at 4 °C. Fourteen 300 μl fractions were manually collected from the top of each gradient. The relative amount of Taf14 and protein standards in the fractions was evaluated by SDS-PAGE and Coomassie brilliant blue total protein staining. 15% PAGE gels were run with Tris-glycine-SDS buffer, except in the case of Taf14ΔN170 analysis, where 16% gel with Tricin-SDS buffer was used due to the small size of this protein. Stained electrophoresis gels were scanned and subjected to a densitometry analysis using Fiji software (42). The apparent molecular weight of the Taf14 proteins was calculated from the determined Stokes radiuses (Rs) and Svedberg sedimentation coefficients (S) using the following formula: Mw(app) = 4.205(SRs) (37).

### DNA electrophoretic mobility shift assay

140 bp *S. cerevisiae VPS13* gene segment was amplified with PCR and purified using GEL/PCR Purification Mini Kit (Favorgen). 0.5 pmol of DNA was incubated on ice for 30 min with increasing amounts (12.5–400 pmol) of purified wildtype or mutant Taf14 proteins in the binding buffer (12.5 mM Hepes-KOH, pH 7.5, 5% glycerol, 50 mM NaCl) in a reaction volume of 15 μl. The reaction mixture was loaded on 5% native polyacrylamide (29:1 acrylamide:bisacrylamide) gel, and electrophoresis was performed in 0.2 × TBE buffer (18 mM Tris, 12.92 mM boric acid, 0.4 mM EDTA) at 130 to 160 V on ice. Gels were stained with SYBRGreen I (Invitrogen; S-7563) and visualized with UVP BioSpectrum Imaging System. The assay was replicated three times. For the calculation of the apparent dissociation constant, gel images were quantified using Fiji (ImageJ2) image processing package by measuring the signal density of free and bound bands. The percentage of bound DNA from the total signal was calculated for each lane and plotted against protein concentration using GraphPad Prism 9.4.0 built-in XY nonlinear regression (curve fit) analysis with an equation with Hill slope.

## Data availability

The mass spectrometry proteomics data have been deposited to the ProteomeXchange Consortium *via* the PRIDE (43) partner repository with the dataset identifier PXD032862.

## Supporting information

This article contains supporting information (30, 44).

## Conflict of interest

The authors declare that they have no conflicts of interest with the contents of this article.
