# Supplementary material for: Transcriptional regulator Taf14 binds DNA and is required for the function of transcription factor TFIID in the absence of histone H2A.Z
Source: J Biol Chem. 2022 Aug 13;298(9):102369. doi: 10.1016/j.jbc.2022.102369 (PMC9478928; doi:10.1016/j.jbc.2022.102369)
Supplement: Supporting Information [file mmc1.pdf]

Supporting Information for:

Transcriptional regulator Taf14 binds DNA and is required for the function of transcription factor TFIID in the absence of histone H2A.Z

Kadri Peil[1], Signe Värv[1], Ivar Ilves[2], Kersti Kristjuhan[1], Henel Jürgens[1], Arnold Kristjuhan[1,3]

[1]Institute of Molecular and Cell Biology, University of Tartu; Riia 23, Tartu 51010, Estonia
[2]Institute of Technology, University of Tartu; Nooruse 1, Tartu 50411, Estonia
[3]corresponding author, arnoldk@ut.ee

Includes:

Table S1. Yeast strains
Figure S1. Deletion of the components of the SWR1 complex is lethal in taf2ΔC1261 cells
Figure S2. Hydrodynamic analysis of the Taf14 proteins

Supplementary Table 1. Yeast strains

Strain	Genotype	Source
AKY152	W303 *MAT A ura3-1 leu2-3,112 his3-11,15 trp1-1 ade2-101 lys2D can1-100*	[1]
AKY1864	*as AKY152, but ADE2*	This study
AKY393	W303 *MAT α, htz1::kanMX*	This study
AKY2124	W303 *MAT α, htz1::natMX*	This study
AKY1819	W303 *MAT A, taf14::kanMX*	[2]
AKY1916	W303 *MAT A, taf14::klURA3*	[2]
AKY2062	W303 *MAT A, taf2::TAF2-3xFlag-natMX*	This study
AKY1900	W303 *MAT A, taf2::taf2Δ1261-1407-3xFlag-kanMX*	This study
AKY2500	W303 *MAT A, taf2::taf2Δ1281-1407-3xFlag-natMX*	This study
AKY1749	W303 *MAT A, taf14::klURA3-3xFlag-TAF14*	This study
AKY1850	W303 *MAT A, taf14::HIS3-taf14ΔN121*	[2]
AKY2406	W303 *MAT A, taf14::HIS3-3xFlag-taf14ΔN145*	This study
AKY2285	W303 *MAT A, taf14::HIS3-3xFlag-taf14ΔN170*	This study
AKY2436	W303 *MAT A, taf14::klURA3-3xFlag-taf14YEATS-spHIS5*	This study
AKY2546	W303 *MAT A*, *taf14::HIS3-3xFlag-taf14ΔN146-170*	This study
AKY2067	W303 *MAT α*, *swr1::natMX*	This study
AKY2139	W303 *MAT α*, *swc5::natMX*	This study

1. Thomas BJ, Rothstein R. Elevated recombination rates in transcriptionally active DNA. Cell. 1989;56(4):619–30.
2. Peil, K., Jürgens, H., Luige, J., Kristjuhan, K., and Kristjuhan, A. (2020). Taf14 is required for the stabilization of transcription pre-initiation complex in Saccharomyces cerevisiae. Epigenetics Chromatin *13*, 24.

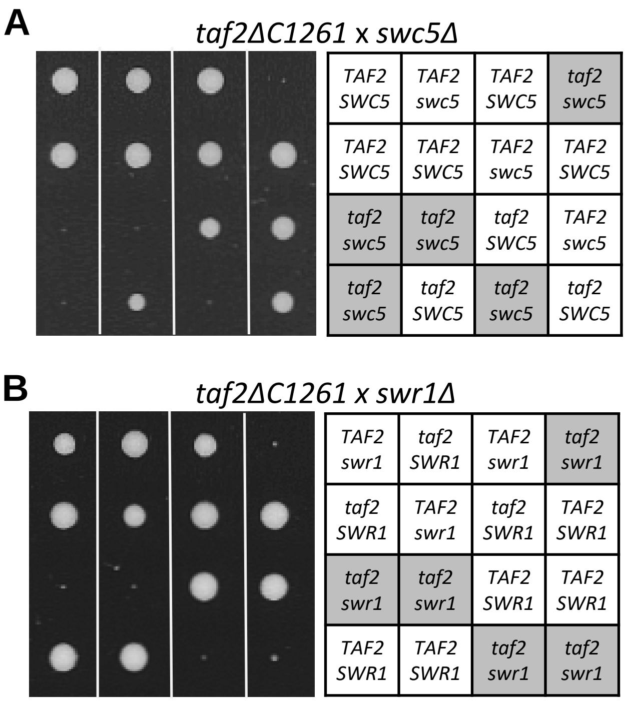

Figure S1. ***Deletion of the components of the SWR1 complex is lethal in taf2ΔC1261 cells.*** Tetrad analysis of the progeny from the crosses between *taf2ΔC1261* and *swc5Δ* (**A**) or *swr1Δ* (**B**). The tetrads were dissected on YPD plates and photographed after 3 days of growth at 30°C. Four tetrads from each dissection are shown. Legends of genotypes are shown on the right panels.

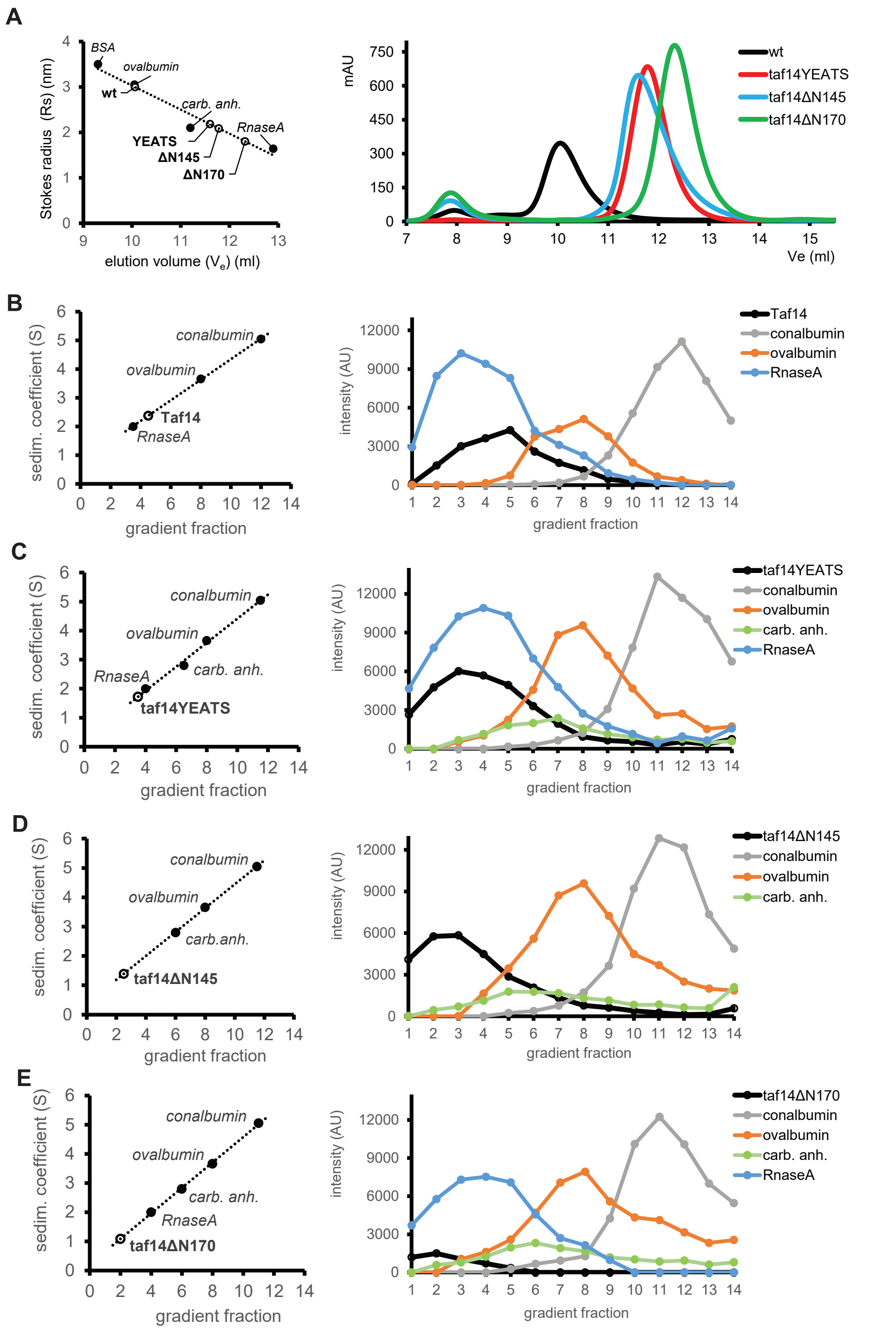

Figure S2. Hydrodynamic analysis of the Taf14 proteins

(A) Size exclusion chromatography analysis of the Taf14 proteins. Right graph shows the overlay of Superdex 75 chromatography profiles, which were used to determine the elution volume of the Taf14 proteins (Ve). In the left graph, the Stokes radiuses that correspond to these Ve values are plotted together with the calibration curve. Following standard proteins with known Stokes radius were used for calibrating the column: BSA (Rs=3.5), ovalbumin (Rs=3.05), carbonic anhydrase (Rs=2.1), and Rnase A (Rs=1.64).

(B)–(E) Glycerol gradient sedimentation analysis of Taf14 proteins. Right graphs show the distribution of indicated Taf14 and standard proteins in the fractions of the glycerol gradient, quantified with the help of densitometry analysis of the Coomassie Brilliant blue stained SDS-PAGE protein gels. In the left graphs, the sedimentation coefficients of the respective Taf14 proteins that correspond to the peak fraction numbers derived from the densitometry analysis are plotted together with the calibration curves. Following standard proteins with known Svedberg sedimentation coefficients were used for the calibration of glycerol gradients: conalbumin ($S_{20,w}$ =5,05), ovalbumin ($S_{20,w}$ =3,66), carbonic anhydrase ($S_{20,w}$ =2,8), and Rnase A ($S_{20,w}$ =2,0) (2.4S). Carbonic anhydrase was omitted from the Taf14 and Rnase A from the Δ N145 gradient run, as these proteins move too close to each other in the SDS-PAGE gel.
